# Mitochondrial Genome Sequences of Four Strains of the Bloom-Forming Raphidophyte *Heterosigma akashiwo*

**DOI:** 10.1128/genomeA.01288-16

**Published:** 2016-12-08

**Authors:** Yoshitoshi Ogura, Natsuko Nakayama, Tetsuya Hayashi, Shoko Ueki

**Affiliations:** a Department of Bacteriology, Faculty of Medical Sciences, Kyushu University, Fukuoka, Japan; b National Research and Development Agency, Japan Fisheries Research and Education Agency, Yokohama, Japan; c Institute of Plant Science and Resources, Okayama University, Okayama, Japan

## Abstract

We report here the complete mitochondrial genome sequences of four strains of bloom-forming raphidophytes from *Heterosigma akashiwo*. These 39-kb sequences contain 42 protein-, two rRNA-, and 26 tRNA-coding sequences. Notable sequence variations were observed among these four newly sequenced and three previously characterized strains, suggesting their potential usage as strain-specific markers.

## GENOME ANNOUNCEMENT

*Heterosigma akashiwo* is a photosynthetic eukaryotic unicellular alga that belongs to the class *Raphidophyceae* and is one of the bloom-causing algae. It is widely identified in temperate thalassic sea of the Pacific-Rim area, including North and South America, eastern Asia, Oceania, and the northern Atlantic area (1–11). Because its bloom may cause significant damages to the fishing industry in the area, its population dynamics in the environment is of great interest and importance. Establishing strain markers will allow monitoring of geographical and temporal dynamics of the species. While the sequence information of *H. akashiwo* microsatellites is potentially of great use as a molecular marker to identify strains, off-target amplification may hamper its usage (12).

Here, as a part of our efforts to establish strain-specific markers for *H. akashiwo*, we sequenced full-length mitochondrial DNA (mtDNA) of four *H. akashiwo* strains obtained from different geographic points: H93616 (obtained from Uranouchi Bay, Kochi Prefecture, Japan), Ha00_17 (Fukuoka Bay, Fukuoka Prefecture, Japan), HaTj01 (Tajiri, Bay, Hiroshima Prefecture, Japan), and NEPCC522 (Jericho Beach, British Colombia, Canada). Total DNA was extracted from *H. akashiwo* cells using a modified cetyltrimethylammonium bromide (CTAB) extraction method (13). The libraries were sequenced by Illumina MiSeq, and the obtained reads were assembled using the *Platanus* software (14). In each strain, two contigs (~30 kb and ~8.5 kb) which covered parts of the previously published mtDNA sequence of *H. akashiwo* strain CCMP452 (15) (accession number GQ222228.1) were obtained. The two gaps in each strain were filled by Sanger sequencing of bridging PCR products. The accuracy of assembly was confirmed by mapping the paired-end reads on the final assemblages using BWA (16). Protein-coding sequence (open reading frame [ORF]) prediction and gene annotation were performed with the mitochondrial gene annotation server (MITOS) (17), ORF Finder, and a database search with BLASTX.

The lengths of the mtDNAs of strains H93616, Ha00_17, HaGS95, HaTj01, and NEPCC522 were 38,764 bp, 38,726 bp, 38,762 bp, and 38,716 bp, and the G+C contents were 35.6%, 35.6%, 35.6%, and 35.6%, respectively. Seventeen respiratory genes (*atp6*, *atp8*, *atp9 cyb*, *cox1*, *cox2*, *cox3*, *nad1*, *nad1l*, *nad2*, *nad3*, *nad4*, *nad4l*, *nad5*, *nad6*, *nad7*, and *nad9*) were identified in each strain, as previously shown in strains CCMP452, NIES293 (accession no. GQ222227), and Y (accession no. NC_016738). Similarly, 26 previously known tRNA genes were identified in all of the 4 strains. Notable sequence polymorphisms were observed among the 7 strains, especially between positions 4350 and 5500, suggesting that *H. akashiwo* mtDNA sequences may provide strain-specific markers that are useful to identify the geographic origins of strains.

### Accession number(s).

The sequences described in this paper have been deposited in DDBJ/EMBL/GenBank under the accession numbers KU561547 (for strain H93616), KU561548 (Ha00_17), KU561550 (HaTj01), and KU726247 (NEPCC522).
